# Interleukin 6 reduces allopregnanolone synthesis in the brain and contributes to age-related cognitive decline in mice

**DOI:** 10.1194/jlr.RA119000479

**Published:** 2020-10

**Authors:** Eileen E. Parks, Sreemathi Logan, Alexander Yeganeh, Julie A. Farley, Daniel B. Owen, William E. Sonntag

**Affiliations:** ^1^Oklahoma Center for Geroscience and Healthy Brain Aging, University of Oklahoma Health Sciences Center, Oklahoma City, OK, USA; ^2^Oklahoma Center for Neuroscience, University of Oklahoma Health Sciences Center, Oklahoma City, OK, USA; ^3^Department of Rehabilitation Sciences, College of Allied Health, University of Oklahoma Health Sciences Center, Oklahoma City, OK, USA; ^4^Department of Biochemistry and Molecular Biology, University of Oklahoma Health Sciences Center, Oklahoma City, OK, USA

**Keywords:** aging, Alzheimer’s disease, inflammation, steroid hormones, enzyme regulation, progesterone, neurosteroid, cognitive function

## Abstract

Cognitive decline with age is a harmful process that can reduce quality of life. Multiple factors have been established to contribute to cognitive decline, but the overall etiology remains unknown. Here, we hypothesized that cognitive dysfunction is mediated, in part, by increased levels of inflammatory cytokines that alter allopregnanolone (AlloP) levels, an important neurosteroid in the brain. We assessed the levels and regulation of AlloP and the effects of AlloP supplementation on cognitive function in 4-month-old and 24-month-old male C57BL/6 mice. With age, the expression of enzymes involved in the AlloP synthetic pathway was decreased and corticosterone (CORT) synthesis increased. Supplementation of AlloP improved cognitive function. Interestingly, interleukin 6 (IL-6) infusion in young animals significantly reduced the production of AlloP compared with controls. It is notable that inhibition of IL-6 with its natural inhibitor, soluble membrane glycoprotein 130, significantly improved spatial memory in aged mice. These findings were supported by in vitro experiments in primary murine astrocyte cultures, indicating that IL-6 decreases production of AlloP and increases CORT levels. Our results indicate that age-related increases in IL-6 levels reduce progesterone substrate availability, resulting in a decline in AlloP levels and an increase in CORT. Furthermore, our results indicate that AlloP is a critical link between inflammatory cytokines and the age-related decline in cognitive function.

Previous studies have clearly indicated that aging is the primary risk factor for cognitive decline and neurodegenerative disease (1–3). Several factors have been implicated in cognitive decline with age, including a decrease in circulating growth factors and neurosteroids and/or an increase in inflammatory cytokines (4). The age-related changes in the levels of neurosteroids are of particular interest because they are produced both in the periphery and the brain, have a complex biochemistry and physiology, and affect multiple pathways involved in cognitive function. Progesterone (PRG) is the precursor for the vast majority of these compounds and enzymatic conversion to allopregnanolone (AlloP), androgens, and corticosterone (CORT) have effects on neurogenesis, synaptic density, and learning and memory (5). AlloP has been shown to be reduced with age and in several neurodegenerative disease models, such as AD and multiple sclerosis (6–8). Restoring the levels of AlloP in these disease models increases neurogenesis, reduces inflammation, and improves cognition (9). Nevertheless, a rigorous analysis of the age-related decline in AlloP and the cellular mechanisms that contribute to the decline in AlloP has not been undertaken.

Previous studies indicate that interleukin 6 (IL-6), an inflammatory cytokine, increases with age and, in the adrenal cortex, upregulates the expression of two enzymes, 11β-hydroxylase and 21-hydroxylase, which convert PRG to CORT (10). These enzymes, as well as others that lead to AlloP synthesis, are found in the endoplasmic reticulum and are expressed throughout various cell types in the brain (11). Because CORT and AlloP share the same precursor, increased synthesis of CORT has the potential to reduce precursor availability for synthesis of other neurosteroids and effectively decrease AlloP levels. Whether similar changes in AlloP and CORT occur in the brain and contribute to the age-related decline in cognitive function is unknown.

The purpose of the present study was 3-fold: *a*) to establish whether neurosteroid levels in brain decrease with age, *b*) to determine whether AlloP replacement improves cognitive function in older animals, and *c*) to assess whether inflammatory cytokines that have been shown to alter steroidogenic enzyme activity in the periphery have a similar effect in the CNS and establish their mechanisms of action. In this study, we investigated the effects of AlloP on cognitive function in aged (24 months) mice as well as the impact of IL-6 on AlloP synthesis. Using LC/MS to quantify the levels of AlloP in the cerebral cortex of male mice, we observed a significant decline with age. Interestingly, administration of a single subcutaneous injection of AlloP improved learning and memory in 24-month-old mice to a level comparable to that in young mice. We also report significant differences in the levels of other steroidogenic enzymes with age in the mouse cerebral cortex. Our data show that the expression and activity of PRG-metabolizing enzymes change with age, likely contributing to the reduced production of AlloP.

## METHODS

### Animals

Male C57BL/6N mice (3, 12, and 24 months old) were obtained from the National Institute on Aging aged rodent colony. C57BL/6J mice were bred in house to generate pups for culture experiments. Animals were housed in the pathogen-free rodent barrier facility at the University of Oklahoma Health Sciences Center as described previously (12, 13). All animals were free of helicobacter and parvovirus and were kept on a 12 h light/12 h dark cycle at 21°C. All mice were group housed except for those undergoing intracerebroventricular infusion surgeries. Mice undergoing surgery were singly housed from the day of surgery until day 14, at which time the mice were euthanized. The mice had access to standard irradiated bacteria-free rodent chow (5053 Pico Lab; Purina Mills, Richmond, IN) and reverse osmosis water ad libitum. All procedures were approved by and followed the guidelines of the Institutional Animal Care and Use Committee of the University of Oklahoma Health Sciences Center.

### Drug treatments and antibodies

AlloP (3α-hydroxy-5α-pregnan-20-one) was purchased from Steraloids, Inc. (Newport, RI). AlloP was reconstituted in 22.5% (w/v) 2-hydroxypropyl-β-cyclodextrin, purchased from Sigma (St. Louis, MO). Soluble membrane glycoprotein (gp)130 (sgp130) was purchased from R&D Systems (#468-MG) and reconstituted in vehicle (0.1% BSA in saline) as described previously (14). For intracerebroventricular injections of IL-6 and sgp130, 1.4 μg was dissolved in 100 μl of vehicle to deliver 100 ng per day via a 14 day osmotic pump, at a pump rate of 0.25 μl per hour (Alzet, mini-osmotic pump, model 1002). BSA (0.1%) in saline was used as the vehicle control for both the sgp130 and IL-6 intracerebroventricular injection studies. IL-6 was purchased from Invitrogen and reconstituted in the vehicle (0.1% BSA in saline). Primary antibodies used for immunoblot analysis included NeuN, Calnexin (Millipore), and Cox 4 (Invitrogen), as well as p-STAT3 and STAT3 (Cell Signaling). Secondary antibodies used included goat anti-mouse IRDye 800 and IRDye 680 (LI-COR) and goat anti-rabbit IRDye 800 and IRDye 680 (LI-COR).

### LC/MS analysis of steroids

PRG), AlloP, and 5α-dihydroprogesterone were analyzed by LC/MS at the Wayne State University Lipidomics core facility. Cerebral cortices from 3-, 12-, and 24-month-old mice were dissected from the brain and flash-frozen under liquid nitrogen prior to processing. C18 extraction columns (30 mg sorbent, 1 ml; Strata-X; Phenomenex, Torrance, CA) were used to isolate steroidal hormones as described earlier for eicosanoids (15, 16). Deuterium-labeled internal standards (progesterone-d9, allopregnanolone-d9, and dihydroprogesterone-d9) were added to the samples prior to analysis via HPLC. HPLC was conducted using a Prominence XR system (Shimadzu) with a Luna C18 (3 μ, 2.1 × 150 mm) column. The mobile phase gradients were: A, water-acetonitrile-formic acid (10:90:0.1 v/v); and B, water-acetonitrile-formic acid (5:95:0.1 v/v). Following HPLC elution, MS was performed using an ESIQTRAP5500 mass analyzer (SCIEX) in the positive ion mode. Unique molecular ions for each steroid analyzed were detected by multiple reaction monitoring. Analyst 1.6.2 software (SCIEX) was used to collect the data, and each multiple reaction monitoring transition chromatogram was quantified by MultiQuant software (SCIEX). Each analyte was quantified following normalization to the internal standards in each chromatogram. Steroid concentrations are expressed as picograms per milligram tissue weight.

### Tissue preparation and solid phase extraction of steroids for ELISAs

Steroids were extracted from brain tissue homogenates as described previously (17). Briefly, each tissue sample was weighed and homogenized in ice-cold ddH_2_O (three times sample volume). Methanol was then added at a volume of four times the homogenate volume (approximately 400 μl) and sonicated at room temperature. The homogenates were then shaken at 1,000 rpm for 1 h at room temperature, and then stored overnight at 4°C. The following day, the homogenates were centrifuged at 3,000 *g* for 10 min at 4°C. The supernatant was collected and diluted with 9 ml of ddH_2_O prior to solid phase extraction .

Bond Elut C18 columns (#12102052) were purchased from Agilent Technologies and used for extraction of steroids. Three milliliters of HPLC-grade ethanol were added to each column. Two minutes later, the columns were then rinsed with 5 ml of ddH_2_O (two times). The samples were then added to the columns at a volume of 5 ml (two times) (10 ml total sample). The columns were again rinsed twice with 5 ml of ddH_2_O, and steroids were extracted with the addition of 2 ml of 90% methanol. The column eluates were then dried under nitrogen at 45°C and stored at −20°C until further use. Prior to ELISA, the samples were reconstituted in sample buffer (provided in both AlloP and CORT ELISA kits).

### ELISAs

The following enzyme immunoassays were used to quantify steroids and proteins in both cortical tissue samples and tissue culture lysates: AlloP and CORT (Arbor Assays), mouse IL-6 (Abcam), mouse 21-hydroxylase (Aviva Systems Biology), and 5-α-reductase (LifeSpan BioSciences). Plates were read at 450 nm wavelength using a SpectraMax M2 plate reader (Molecular Biosystems). Sample concentrations were determined using a standard curve of the standards provided in each kit. Steroid concentrations were quantified as picograms per milligram of tissue weight, and protein levels were quantified as picograms per milligram of total protein.

### RAWM spatial memory assessment

To assess the effects of AlloP on spatial memory in aged mice, AlloP was subcutaneously injected into aged mice at 10 mg/kg body weight in a volume of 100 Ul. 2-Hydroxypropyl-β-cyclodextrin was used as the vehicle for both young and aged control groups and given at a volume of 100 ul. Both AlloP and vehicle injections were administered 10 days prior to behavioral testing. The radial arm water maze (RAWM) was used to measure spatial learning and memory. The RAWM consists of an eight-arm maze in which the mouse swims from a starting arm to the platform in the target arm to escape the water, as previously described (13). Briefly, each mouse underwent eight (60 s) trials per day over three consecutive days. Movement of mice in the maze was detected by a video tracking system located above the maze and parameters were measured using Ethovision software version 10 (Noldus Information Technology Inc., Leesburg, VA) as described previously (18). Performance was measured by velocity, latency (time taken to reach the target platform), and errors (entries into incorrect arms). Data were analyzed using JMP statistical software (SAS Institute, Inc., Cary, NC).

### Spatial memory assessment using PhenoTyper with CognitionWall

Spatial memory was assessed following vehicle (0.1% BSA in saline) or sgp130 intracerebroventricular infusion using the PhenoTyper with CognitionWall (Model 3000; Noldus Information Technology, The Netherlands). Sgp130 was delivered intracerebroventricularly at 100 ng/day over 14 days. The mice began behavioral testing on day 10 post intracerebroventricular infusion surgery, and completed testing on day 14, after which the mice were euthanized. To familiarize the mice with the precision rodent pellets used in the PhenoTyper, each mouse was singly housed for 4 days prior to testing with access to traditional rodent chow and precision rodent pellets ad libitum. The PhenoTyper home cage testing apparatus was used to assess initial discrimination learning and reversal learning as described previously (12). During initial discrimination, the mouse must enter the left of three entrances five times (FR5) to receive a food pellet. During the reversal period, the mouse must enter the right entrance five times to receive a food pellet. Activity of the mice was recorded using Ethovision software version 11 (Noldus).

### Tissue preparation

After completion of the experiments, mice were euthanized by cervical dislocation and both hemispheres of the brain were dissected, frozen in liquid nitrogen, and later used for mRNA expression analysis, protein quantification, or steroid extraction.

### Microsome isolation

Microsomes containing the endoplasmic reticulum were isolated from a separate cohort of animals as described previously (19). Mice (3, 12, and 24 months old) were euthanized and the cerebral cortex was dissected and homogenized in sucrose buffer [250 mM sucrose, 5 mM HEPES-KOH (pH 7.4), 1 mM EGTA, 1 mM DTT, and protease inhibitors]. Tissues were homogenized on ice using a dounce homogenizer. To remove the nuclear fraction, the homogenates were centrifuged at 500 *g* for 15 min. The supernatant was then collected and centrifuged at 8,000 *g* for 15 min to remove the mitochondrial fraction. Finally, the supernatant containing the microsomal fraction was transferred to a new tube and diluted with 125 mM sucrose, followed by centrifugation at 21,000 *g* for 90 min. The resulting pellet containing the microsomes was then resuspended in storage buffer [250 mM sucrose, 5 mM HEPES-KOH (pH 7.4), 1 mM EGTA, protease inhibitors, and 30% glycerol] and kept at −80°C until future use.

### PRG metabolism assays

Microsomes were rinsed in sodium phosphate buffer (0.01 mol/l, pH 7.0) containing protease inhibitors. For all incubations, 150 μg of protein were suspended in 1 ml of sodium phosphate buffer containing NADPH (10^−3^ mol/l) and 0.1 mCi/ml 4-C^14^-PRG (10 nM), as described previously (20). Following analysis of the time course and dose response curves, we determined the optimum incubation conditions to be 60 min using 10 nM 4-C^14^-PRG. The reaction was incubated at 37°C in a shaking water bath. After 60 min, the reaction was stopped by placing the samples on ice and adding 1 ml of ice-cold methylacetate.

### Extraction of PRG metabolites

Each sample was centrifuged at 1,000 *g* for 10 min at 4°C. The upper phase containing the steroid metabolites was transferred into a new tube. Methyl acetate (700 ul) was then added to the remaining lower fractions and resuspended, followed by centrifugation at 1,000 *g* for 10 min. The upper phase was again transferred to a separate tube and rinsed three more times. The collected upper phases containing the steroid fractions were combined and evaporated under a gentle stream of N_2_ gas and stored at −20°C until future use.

### TLC

Steroid fractions were dissolved in methanol and resolved using TLC plates (TLC Silica gel 60 F_254_ glass plates 20 × 20 cm; EMD Millipore) (20). To visually confirm the location of the individual metabolites, a solution of nine reference standards [PRG (Q2600), 5α-dihydroprogesterone (P2750), 20α-dihydroprogesterone (Q3600), 17-hydroxyprogesterone (Q3360), deoxycort (Q3460), CORT (Q1550), epiallopregnanolone (P3830), AlloP (P3800), and testosterone (A6950) (Steraloids)] (10 ug each) dissolved in methanol were pipetted onto the individual plates at a volume of 10 μl. Each sample was run on an individual TLC plate containing the reference standards. Each plate was run two-dimensionally using methyl acetate-ethylene dichloride (65:35) for one direction and hexanol-hexane (75:25) for the other direction. The plates were dried overnight and then stained with an aqueous solution of 3% cupric acetate and 8% phosphoric acid to visualize the location of reference standards. The plates were then dried for 30 min at 110°C. Individual spots (Fig. 4B) containing each ^14^C-labeled metabolite were identified based on their migration on the plate, scraped from the plate, and transferred to scintillation vials. Each spot was quantified by scintillation counting and specific activity calculated as picomoles per milligram per hour.

### Gene expression

Total RNA was isolated from hippocampus and cortex using the Qiagen RNeasy kit and reverse transcribed using a high-capacity RNA-to-cDNA kit (Applied Biosystems). TaqMan primers were purchased from Thermo Fisher. Gene expression of steroidogenic enzymes (5α-reductase type 1) [Mm00614213], 21-hydroxylase [Mm00487230], 17α-hydroxylase [Mm00484040], and IL-6 (Mm00446190) were quantified using the Quantstudio 12K Flex System (Applied Biosystems, Life Technologies, Waltham, MA) using TaqMan universal master mix reagents (Thermo Fisher). All data were normalized to the housekeeping genes, HPRT [Mm03024075_m1] and GAPDH [Mm99999915_g1] and presented as percent mRNA expression relative to young control groups.

### Western blotting

Tissues and cell culture lysates were homogenized in lysis buffer (RIPA Lysis Buffer, Thermo Fisher) containing protease inhibitors, followed by sonication and centrifugation at 15 000 *g*. The supernatants containing the soluble proteins were quantified by the Bio-Rad detergent compatible protein assay and denatured with 1% SDS. Proteins were separated by SDS-PAGE and transferred to nitrocellulose membranes. Immunoblots were probed with the appropriate primary antibodies, rinsed, and then probed with fluorescent secondary antibodies (IRDye 800CW or IRDye 600CW) (LI-COR Biosciences) as described previously (21). An Odyssey infrared imaging system (LI-COR Biosciences) was used to image and quantify all protein bands.

### Cell cultures

Primary mouse cortical neurons and astrocytes were used in the in vitro experiments. Cortical neurons were isolated from E18-E20 C57BL/6J mouse pups according to approved Institutional Animal Care and Use Committee guidelines. Cortical astrocytes were isolated from postnatal days 2–4 C57BL/6J mouse pups. Astrocytes and neurons were isolated as described (13, 21). Briefly, following papain enzymatic digestion and trituration, cortical cells were resuspended in growth media [neurobasal medium containing 2% NuSerum (BD Biosciences), 2% B27 (Life Technologies), penicillin/streptomycin (10 μg/ml), and L-glutamine (29.2 μg/ml)]. Neurons were seeded on 50 mg/ml poly-D-lysine-coated 6-well plates at a density of 2 × 10^5^ cells/well and half of the media was replaced with fresh media every 2–3 days. After 72 h of incubation, the neuronal cultures were treated with 5-fluor-2′-deoxyuridine (1.5 μg/ml) and uridine (3.5 μg/ml) for inhibition of astrocyte proliferation. For astrocyte cultures, cells were seeded on 50 mg/ml poly-D-lysine-coated 10 cm^2^ dishes at a density of 1.5 × 10^6^ cells/dish. After 7 days in vitro, cells were split with 0.25% Trypsin-EDTA and seeded on 50 mg/ml poly-D-lysine-coated 6-well plates at a density of 2 × 10^5^ cells/well. For IL-6 experiments, drug administration began on day 10 for neurons and day 3 for astrocytes. Astrocyte and neuron cultures were treated with vehicle (0.1% BSA in saline) or 100 ng/ml IL-6 (per well in a 6-well plate) for 24 h, after which the lysates were harvested for mRNA and protein analyses. For cell culture AlloP and CORT analyses, the cells were treated with vehicle or 100 ng/ml IL-6 for 24 h, followed by the addition of 10 nM PRG for 1 h. Cell culture medium was then harvested for neurosteroid analysis. The cells were maintained at 37°C with 5% CO_2_ in a tissue culture incubator.

### Statistics

Systat (version 11; Systat Software, San Jose, CA), JMP statistical software package (JMP Pro Version 14.1, SAS Institute), and GraphPad Prism were used for data analysis and graphing. LC/MS and gene expression data were quantified using a one-way or two-way ANOVA, as appropriate. Post hoc comparisons were made using the Holm-Sidak method. Data collected for analysis of cognitive function were analyzed using treatment group, task (initial discrimination or reversal), hour and phase (day or night) as independent variables. RAWM data were also analyzed with treatment group, task, trial, and day as independent variables. Significant differences in main effects or their interactions were analyzed using the Tukey HSD for pairwise comparisons. All data are represented as the mean ± SEM.

## RESULTS

### AlloP levels are altered with age

**Fig. 1.**
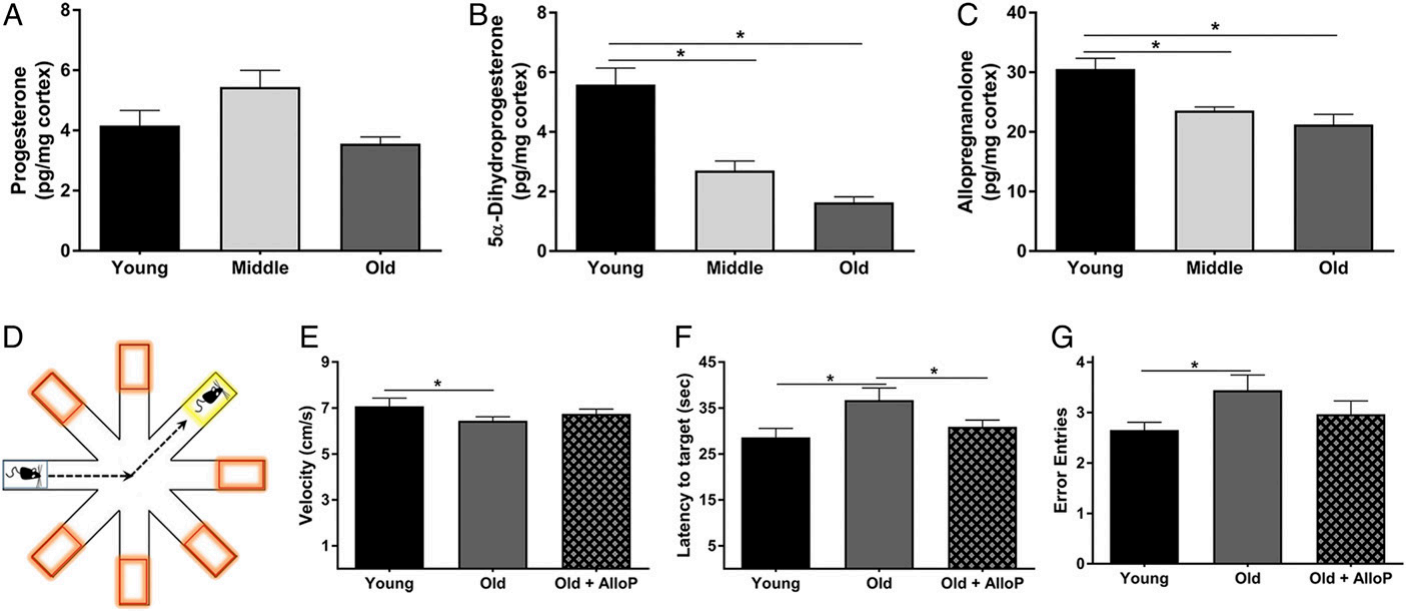
AlloP decreases in the brain with age, and its restoration improves working memory. A–C: LC/MS quantification of PRG (A), 5α-dihydroprogesterone (B), and AlloP (C) in the cerebral cortex from young (3 months), middle-aged (12 months), and old mice (24 months) (n = 6 per group; one-way ANOVA). D: Illustration of the RAWM. Orange boxes indicate incorrect arms, and the yellow box indicates the target arm containing the escape platform. E–G: Performance in the RAWM in young (3 months), old (24 months), and old mice treated with AlloP. E: Average velocity of the mice in each group while in the RAWM. F: Average latency from the starting arm to the target platform. G: Average entries into incorrect arms. E–G: Data represent the average of eight trials per day over 3 days (n = 9 per group; two-way ANOVA and Tukey HSD pairwise comparisons). *P < 0.05. Statistics were adjusted for multiple comparisons. Only significant differences are depicted in the figure. Data are presented as mean ± SEM.

AlloP is synthesized from PRG via two enzymatic steps. It can be synthesized locally in the brain or can easily cross the blood-brain barrier after production by peripheral endocrine organs. To quantify the levels of AlloP and its upstream metabolites with age, we performed LC/MS analysis of cortical brain tissue from young (3 months), middle-aged (12 months), and old (24 months) male mice. Interestingly, PRG levels were not significantly different with age (**Fig. 1A**). In contrast, the levels of 5-α-dihyroprogesterone (*P* = 0.009) and AlloP (*P* = 0.001) decreased at both middle age and old age (Fig. 1B, C).

### AlloP improves working memory in 24-month-old mice

In addition to the reduction of AlloP in neurodegenerative models, previous studies have also shown that restoration of AlloP in young AD mice improves learning and memory. To determine whether restoring AlloP in aged mice could improve cognition, we assessed spatial memory following AlloP administration. A single subcutaneous injection of AlloP (10 mg/kg) or vehicle (β-cyclodextrin) was administered to young and aged mice and, after 10 days, spatial memory was assessed using the RAWM (Fig. 1D). As expected, young mice made fewer entries into incorrect arms than aged control mice (*P* < 0.05, Fig. 1G). Interestingly, AlloP-treated aged mice also performed better than control aged mice and showed similar performance to young mice. Both young and AlloP-treated aged mice had a shorter latency to platform compared with aged control mice (*P* < 0.05, Fig. 1F). These differences were not due to differences in swimming velocity. Thus, a single injection of AlloP is sufficient to improve spatial learning in advanced age mice.

### Neurosteroid synthesis is altered with age

**Fig. 2.**
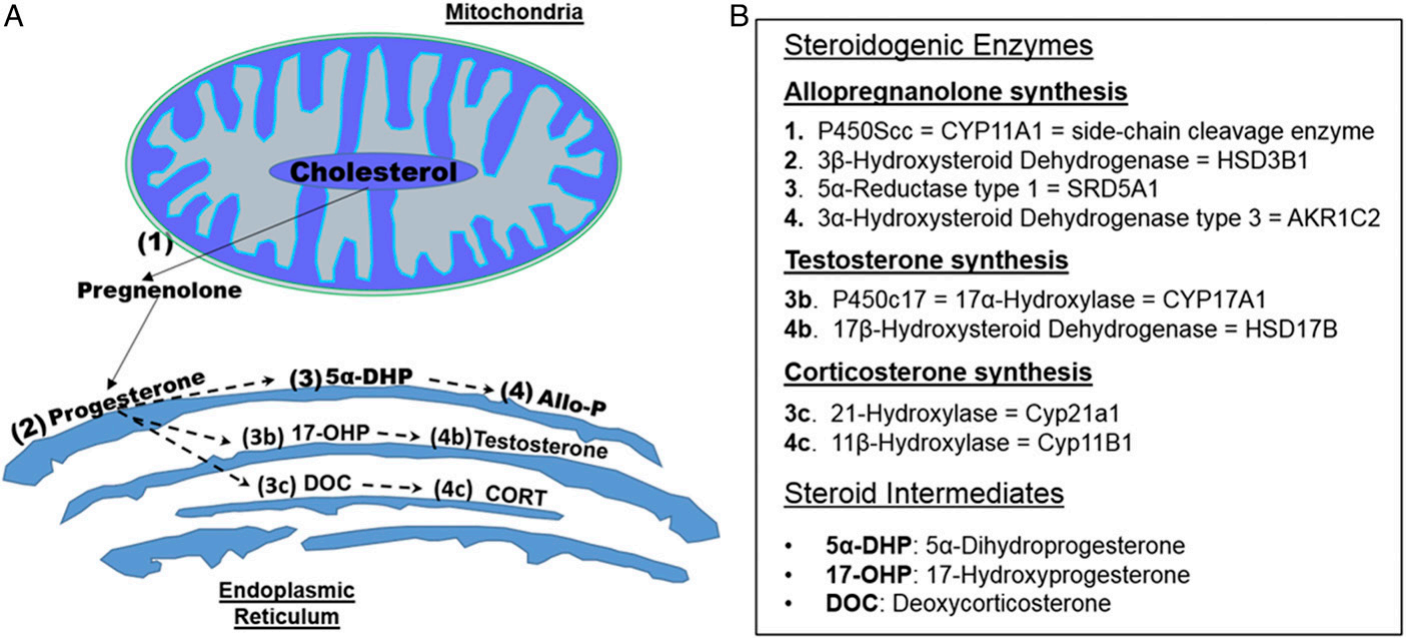
Biosynthetic pathways leading to neurosteroid synthesis. A: Illustration of steroidogenic enzymes produced in the mitochondria and endoplasmic reticulum leading to the synthesis of AlloP, testosterone, and CORT. B: Table outlining the steroidogenic enzymes associated with the respective numbers in A. Cholesterol is the precursor for all neurosteroid synthesis. Cholesterol is first metabolized by P450Scc (1) housed in the mitochondria to form pregnenolone. After exiting the mitochondria, pregnenolone can then be metabolized by 3β-hydroxysteroid dehydrogenase (2), located in the endoplasmic reticulum, to form PRG. PRG can then be metabolized by several different enzymes to form unique steroid metabolites. AlloP is formed via 5α-reductase (3) and 3α-hydroxysteroid dehydrogenase (4), respectively. Testosterone can be formed via P450c17 (3b) and 17β-hydroxysteroid dehydrogenase (4b), respectively. CORT can also be formed from 21-hydroxylase (3c) and 11β-hydroxylase (4c), respectively.

**Fig. 3.**
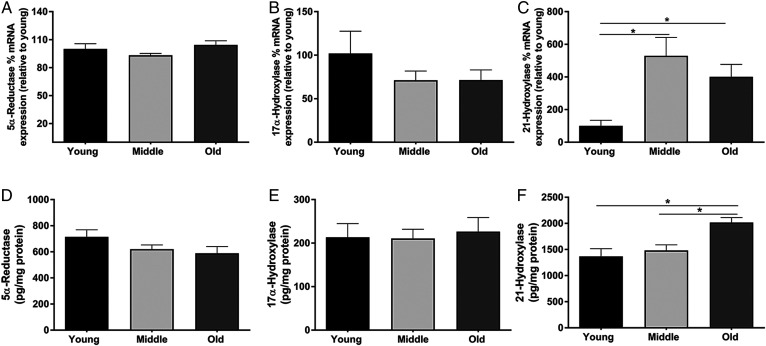
The mRNA expression and protein levels of steroidogenic enzymes are altered with age. A–C: Percent mRNA expression of 5α-reductase, 17α-hydroxysteroid dehydrogenase, and 21-hydroylase in the cortex of young (3 months), middle-aged (12 months), and old (24 months) mice (n = 8 per group; one-way ANOVA). D–F: Protein levels (picograms per milligram of protein) of 5α-reductase, 17α-hydroxysteroid dehydrogenase, and 21-hydroylase in the cortex of young (3 months), middle-aged (12 months), and old (24 months) mice (n = 8 per group; one-way ANOVA). Statistics were adjusted for multiple comparisons. Only significant differences are depicted in the figure. Data are presented as mean ± SEM

The production of AlloP is dependent on the expression and activity of multiple steroidogenic enzymes (**Fig. 2**). Considering that PRG can be metabolized into several other neurosteroids, it is possible that precursors are shunted to alternative pathways leading to the reduction in AlloP. We first quantified the expression of the initiating enzymes that metabolize PRG. Interestingly, the mRNA expression of 5α-reductase, which initiates the production of AlloP, or 17-hydroxyprogesterone did not change with age (**Fig. 3A**, B). However, the expression of 21-hydroxylase, which leads to the synthesis of CORT was significantly upregulated at both middle (*P* = 0.003) and old age (*P* = 0.042) compared with young age (Fig. 3C). To further investigate the hypothesis that increased 21-hydroxylase contributed to the reduction in AlloP synthesis with age, we next quantified the protein levels of PRG-metabolizing enzymes. Similar to the mRNA expression levels, protein levels of 5-α-reductase and 17α-hydroxylase were not changed with age (Fig. 3D, E), while those of 21-hydroxylase were significantly upregulated with age (Fig. 3F).

**Fig. 4.**
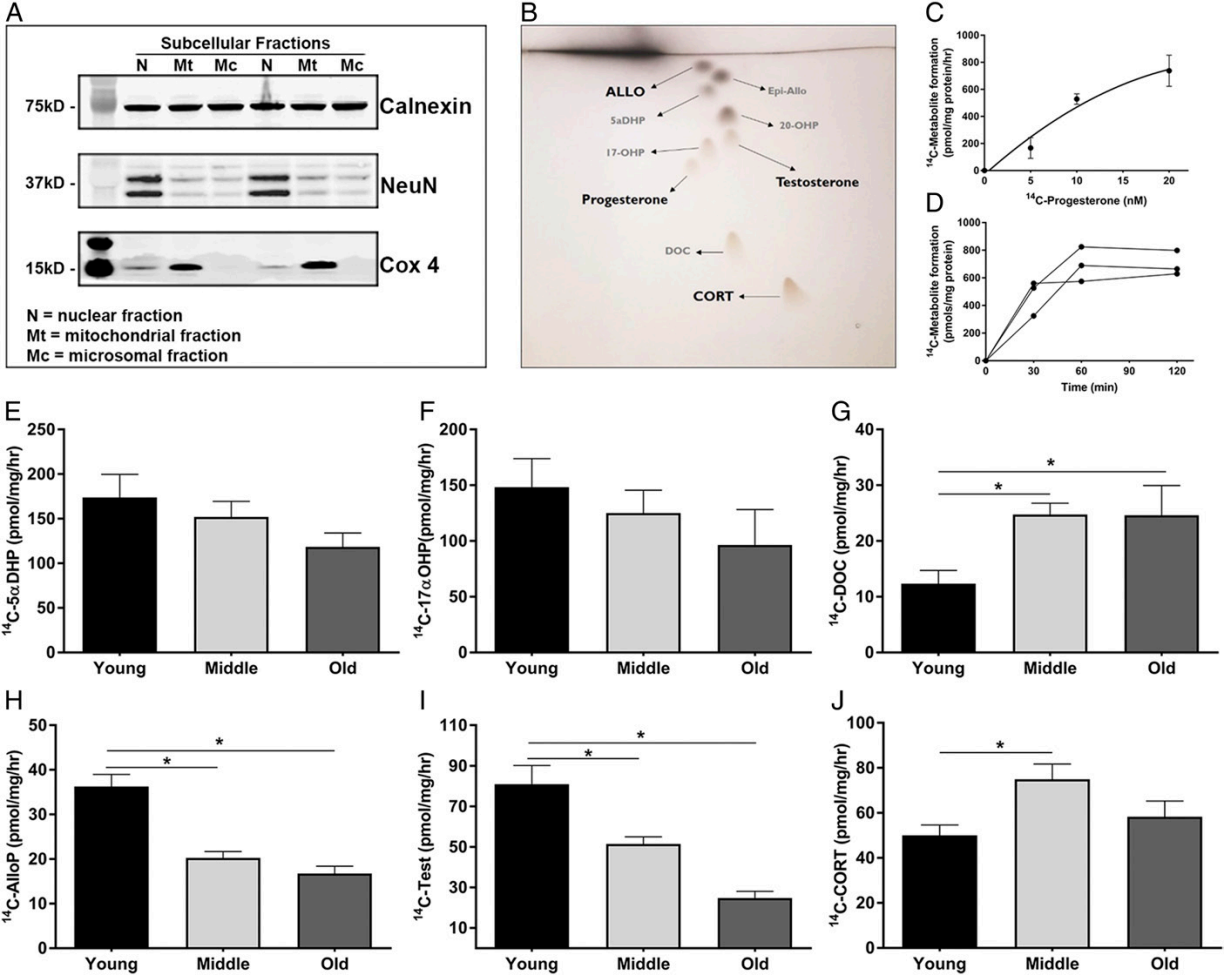
PRG metabolism via steroidogenic enzymes are altered with age in the cerebral cortex. A: Representative Western blot showing the separation of subcellular fractions. Calnexin is a marker for the microsomal fraction, Cox4 marks the mitochondrial fraction, and NeuN marks the nuclear fraction (n = 3, with three replicate experiments). B: Representative image of the TLC plates used to separate the individual steroid metabolites. C: Dose response curve of increasing amounts ^14^C-PRG to ^14^C-metabolites in microsomes (n = 3). Data represent the average total ^14^C-metabolite picomoles formed per milligram of protein per hour. D: Time course of ^14^C-metabolite formation in microsomes following the addition of ^14^C-PRG (n = 3). E–J: Metabolite formation of 5α-dihydroprogesterone (5αDHP) (E), 17α-hydroxyprogesterone (17αOHP) (F), deoxycorticosterone (DOC) (G), AlloP (H), testosterone (Test) (I), and CORT (J) in microsomes isolated from the cerebral cortex of young (3 months), middle-aged (12 months), and old (24 months) mice. PRG metabolite formation is shown as the picomoles of each ^14^C-metabolite formed per milligram of microsomal protein per hour (n = 4 per group; one-way ANOVA) (*P < 0.05). Statistics were adjusted for multiple comparisons. Only significant differences are depicted in the figure. Data are presented as mean ± SEM

To assess the activity of PRG-metabolizing enzymes with age, microsomes containing the endoplasmic reticulum were isolated from the mouse brain cortex via subcellular fractionation using a sucrose gradient. Validation of an enriched microsomal fraction was determined by the absence of the mitochondrial marker, cox 4, and the nuclear marker, NeuN, from the microsomal fraction (**Fig. 4A**). We assessed PRG metabolite formation at 60 min after addition of 10 nM ^14^C-PRG (Fig. 4C, D). The assay revealed that PRG metabolism into both testosterone and its precursor, 17-hydroxyprogesterone, declined with age (Fig. 4 F, I) (*P* = 0.002), as well as AlloP and its precursor 5a-DHP (Fig. 4 E, H) (*P* = 0.042). In contrast, the metabolism of PRG into CORT and its precursor, DeoxyCort, significantly increased with age (Fig. 4 G, J) (*P* = 0.014).

### IL-6 increases in the brain with age and modulates neurosteroid synthesis and working memory

**Fig. 5.**
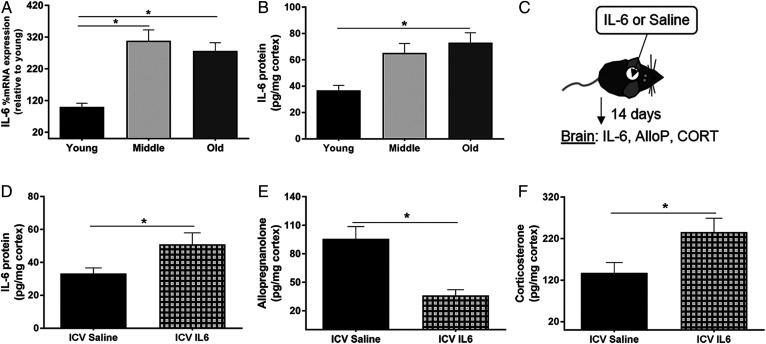
IL-6 increases in the brain with age, and its administration to young mice reduces AlloP synthesis and increases CORT synthesis. A, B: Cerebral cortex tissues from young (3 months), middle-aged (12 months), and old (24 months) mice. A: Percent mRNA expression of IL-6 across age relative to young (n = 8 per group; one-way ANOVA). B: Levels of IL-6 (picograms per milligram of protein) in the cortex across age (n = 6 per group; one-way ANOVA). C: Experimental design of saline or IL-6 infusions to the mouse brain. Saline or IL-6 was loaded into an osmotic pump that was coupled to a cannula and inserted into the lateral ventricle of the brain in young (4 months) mice. The osmotic pump delivered saline or IL-6 (100 ng/day) continuously for 14 days. D: Levels of IL-6 (pg/mg protein) in the cortex following Saline or IL-6 intracerebroventricular infusion. E, F: AlloP and CORT levels in the cortex of young mice following 2 weeks of intracerebroventricular infusions of either saline or IL-6 (100 ng/day) to the lateral ventricle of the brain (n = 8 per group, unpaired Student’s *t*-test, *P < 0.05). Statistics were adjusted for multiple comparisons. Only significant differences are depicted in the figure. Data are presented as mean ± SEM.

Inflammation is known to increase throughout the body with age, and IL-6 has been implicated in the regulation of steroidogenic enzyme expression in the HPA axis (10, 22). To determine whether IL-6 had a role in the age-related changes in enzyme activity and expression, we quantified mRNA expression and protein levels of IL-6 in the cortex of young, middle-aged, and old mice. In accordance with previous literature, mRNA expression and protein levels of IL-6 increased with age (*P* = 0.001 and *P* = 0.017, respectively) (**Fig. 5A**, B) (2). To determine whether IL-6 administration to young mice could suppress AlloP production, we administered IL-6 directly into the lateral ventricle via intracerebroventricular infusion for 14 days. At the end of the 14 day period, the mice were euthanized and the cerebral cortices were isolated for analysis (Fig. 5C). The levels of AlloP were significantly reduced in the cerebral cortex of IL-6-treated mice (*P* = .002) (Fig. 5E), while those of CORT were increased (*P* = 0.034) (Fig. 5F), suggesting that IL-6 regulates AlloP and CORT synthesis in the brain.

**Fig. 6.**
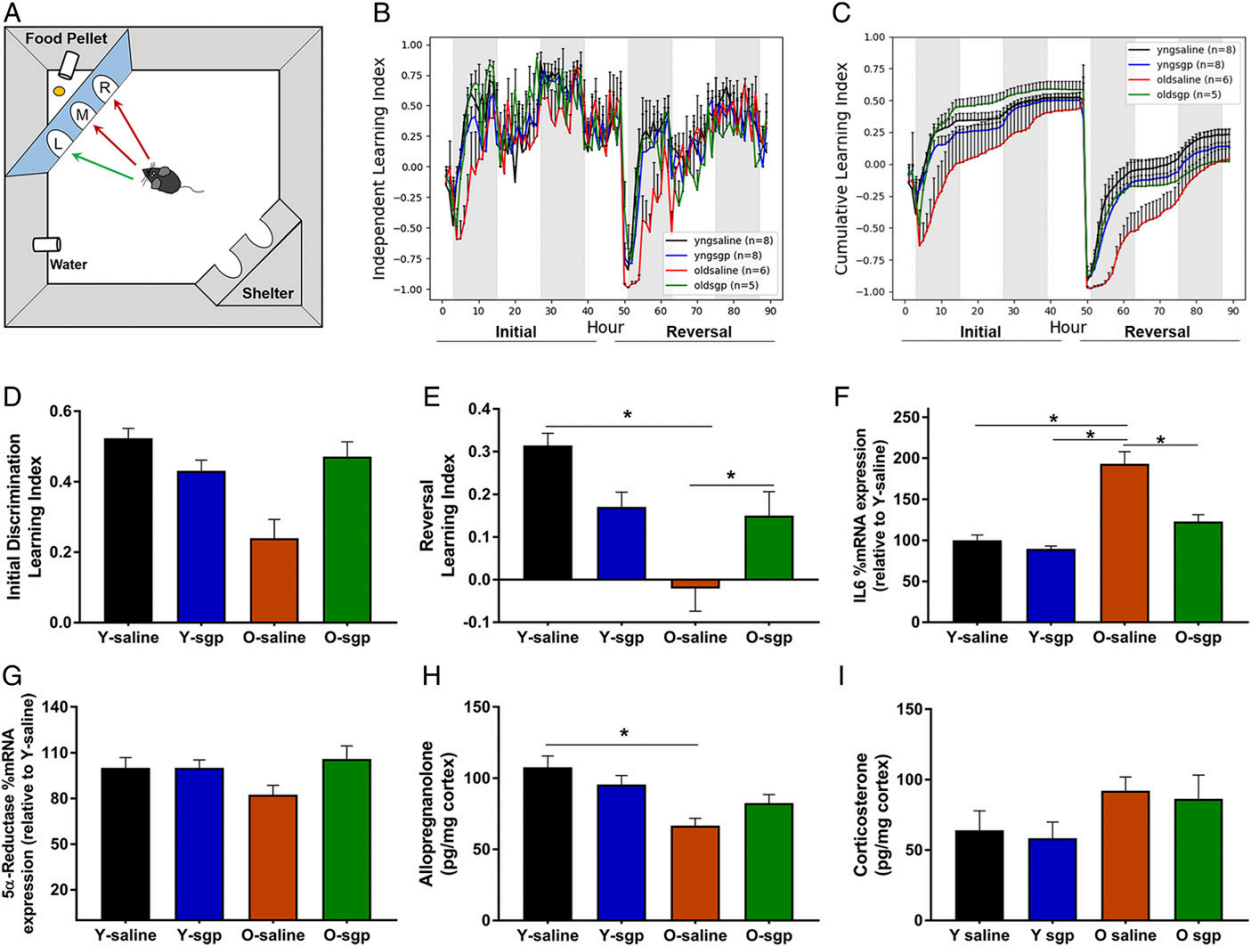
The effects of IL-6 inhibition on neurosteroid synthesis and working memory. A: Illustration of the PhenoTyper home cage system containing the cognition wall used to assess working memory. On days 1–2, the initial learning period, the mouse must learn to enter the left most entry of the cognition wall at least five times to receive a food pellet. On days 3–4, the reversal period, the mouse learns to enter the right most entry at least five times to receive a food pellet. B, C: Working memory assessment in young and old mice following a 2 week intracerebroventricular infusion of either saline or sgp130 (IL-6 inhibitor) to the lateral ventricle of the brain. Learning indices are calculated by (correct entries – incorrect entries)/total entries made per hour. B: The independent index represents the average learning index per group across each hour of the initial learning and reversal periods. C: The cumulative index represents the average learning index per group accumulated over consecutive hours in both the initial learning period and the reversal period. D: Average learning indices per group during the initial discrimination period. E: Average learning indices per group during the reversal period (n = 5–8 per group, two-way ANOVA, Tukey HSD pairwise comparisons, *P < 0.05). F–I: Cerebral cortex tissues from young and old mice treated with saline or the IL-6 inhibitor, sgp130 (n = 5–8 per group, two-way ANOVA, *P < 0.05). F: Percent mRNA expression of IL-6 across groups relative to young. G: Percent mRNA expression of 5α-reductase across groups relative to young. H, I: AlloP and CORT levels in the cortex following saline or sgp130 treatment. Statistics were adjusted for multiple comparisons. Only significant differences are depicted in the figure. Data are presented as mean ± SEM.

To further investigate the effects of IL-6 on steroidogenic enzyme expression and working memory in old mice, we administered the IL-6 inhibitor, sgp130, locally to the brain. Sgp130 was administered to the lateral ventricle of young and aged mice via intracerebroventricular infusion using an osmotic pump. After 14 days, the mice were assessed for spatial memory. Using the cognition wall as previously described (12) (**Fig. 6A**), we assessed the independent learning index (Fig. 6B), which represents the average learning index per group across each hour of the initial and reversal periods. We also assessed the cumulative learning index (Fig. 6C), which represents the average learning index per hour accumulated over consecutive hours in the initial and reversal periods. Although there were strong trends for aged saline groups performing poorly, and aged sgp130 groups performing better during the initial discrimination period (Fig. 6D), the results were not significant. However, during the reversal period, aged control mice performed significantly worse than both young mice treated with sgp130 and young mice treated with saline. Aged mice treated with sgp130 for 14 days had significantly improved working memory compared with age-matched controls (*P* < 0.003) (Fig. 6E). Additionally, area under the curve analyses of the cumulative learning index revealed a significant difference between young (102.6 ± 1.126) and aged (76.6 ± 2.78) control mice (*P* < 0.0001) as well as between aged control (76.6 ± 2.78) and aged mice treated with sgp130 (100 ± 2.32) (*P* < 0.0001) when assessed by two-way ANOVA. Young mice treated with sgp130 showed no differences compared with young control mice. These results suggest that inhibiting IL-6 signaling in aged animals could be a therapeutic target for treating or preventing cognitive decline.

Aged mice treated with sgp130 had significantly reduced IL-6 mRNA expression compared with control aged mice (Fig. 6F), while young mice were unaffected by sgp130 treatment. This is in accordance with our previous data showing lower levels of IL-6 in young mice, potentially making them less likely to respond to IL-6 inhibition. Similar to our previous results, 5α-reductase was not significantly different across age; however, there was a trend for an increase in 5α-reductase in response to sgp130 treatment in aged mice (Fig. 6G). As we observed in our earlier LC/MS data, AlloP was reduced with age (*P* = 0.043). Although there was a trend for an increase in the levels of AlloP in sgp130-treated mice, the results were not significantly different (Fig. 6H). The levels of CORT increased with age, but statistical analysis indicated no significant differences between groups (Fig. 6I).

### IL-6 regulates enzyme activity and controls AlloP synthesis specifically in astrocytes

**Fig. 7.**
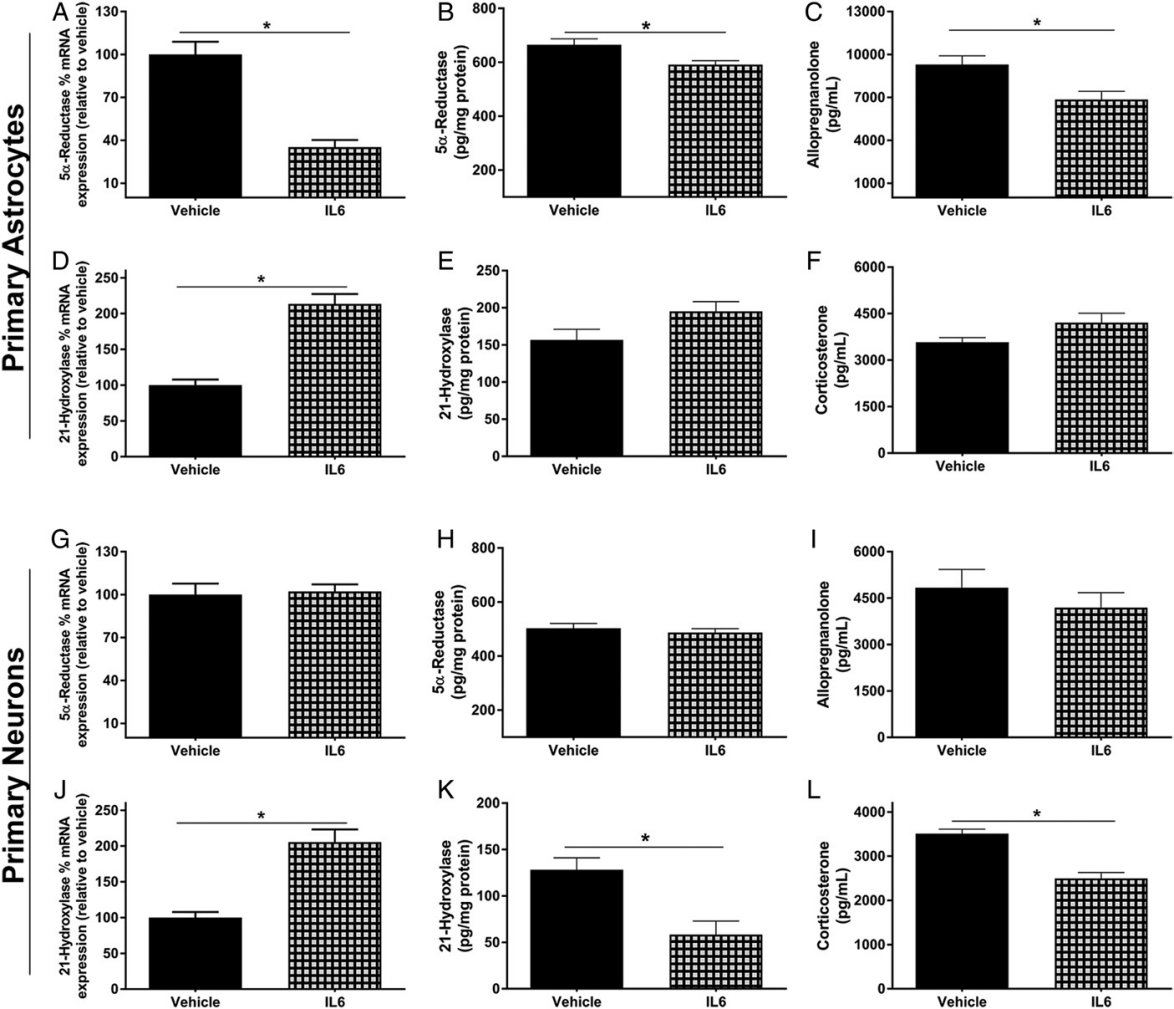
IL-6 reduces the synthesis of AlloP specifically in astrocytes and reduces the synthesis of CORT specifically in neurons. Primary astrocyte and neuronal cultures were treated with or without IL-6 (100 ng/ml) for 24 h and used for the following experiments. Cell culture lysates were used for percent mRNA expression (relative to vehicle control) and protein levels of 5α-reductase and 21-hydroxylase. Cell culture medium was used for AlloP and CORT quantification. 5α-Reductase mRNA expression in astrocytes and neurons (A, G), 5α-reductase protein levels in astrocytes and neurons (B, H), AlloP in cell culture media from astrocytes and neurons (C, I), 21-hydroxylase mRNA expression in astrocytes and neurons (D, J), 21-hydroxylase protein levels in astrocytes and neurons (E, K), CORT in cell culture media from astrocytes and neurons (F, L) (n = 4 replicate experiments; Student’s unpaired *t*-test; Bonferroni method for multiple comparisons, *P < 0.05). Data are presented as mean ± SEM.

To assess the cell-specific effects of IL-6 on steroidogenic enzyme expression and neurosteroid synthesis, we performed in vitro studies using primary neuronal cultures from embryonic day 19-20 pups, and primary astrocyte cultures from postnatal day 3 pups. Astrocytes treated with IL-6 had significantly lower mRNA expression and protein levels of 5α-reductase (**Fig. 7A**, B) (*P* < 0.001), higher mRNA expression of 21-hydroxylase (Fig. 7D) (*P* = 0.001), and a trend for increased 21-hydroxylase protein levels (Fig. 7E). Due to the differences in enzyme expression, we next quantified the production of both AlloP and CORT in primary astrocytes. Twenty-four hours following IL-6 or vehicle treatment, 10 nM of PRG were added as an initiating substrate. After 4 h, the cell culture medium was harvested and steroids were extracted. IL-6 significantly reduced the production of AlloP (*P* = 0.015) and partially increased the production of CORT, although the later effect was not significant (Fig. 7C, F). In contrast, primary neuron cultures had no reduction in mRNA expression or protein levels of 5α-reductase following IL-6 treatment (Fig. 7G and H). Nevertheless, IL-6 did increase the mRNA expression of 21-hydroxylase in primary neurons (*P* = 0.003), similar to that in astrocytes (Fig. 7J). Conversely, IL-6 treatment reduced the protein levels of 21-hydroxylase (*P* = 0.02) (Fig. 7K).When the production of AlloP and CORT in neuronal cultures was quantified, IL-6 had no effect on the production of AlloP (Fig. 7I). Interestingly, in contrast to the increased expression of 21-hydroxylase, IL-6 significantly reduced the production of CORT in primary neurons (*P* = 0.012) (Fig. 7L). These data suggest that IL-6 may play unique roles in different cell types in the brain.

## DISCUSSION

Previous studies have shown that AlloP declines in neurodegenerative disease models (6, 23). However, there have been no studies beyond the age of 14 months (23). Considering that male C57BL/6 mice have an average lifespan of 28 months, 14 months is widely considered to be middle-aged (24, 25). We sought to determine the levels of AlloP in 24-month-old mice, which is more indicative of physiological aging and is consistent with the decline in cognitive function that is generally observed beginning at 21 months of age (26–28). To our knowledge, we are the first to demonstrate that AlloP declines in 24-month-old mice, and more importantly, that replacement of AlloP can improve learning and memory in advanced age.

The mechanism by which AlloP declines with age has not been well studied. Like all steroid hormones, the synthesis of AlloP is dependent on the expression and activity of multiple steroidogenic enzymes in the brain (29, 30). Using microsomes isolated from the cerebral cortex, we found that PRG metabolism into both AlloP and testosterone was reduced with age, while PRG metabolism into CORT was elevated. Thus, the elevation of CORT synthesis with age could be a contributing factor to the reduction in AlloP due to limitations of precursor availability.

The expression patterns of a variety of steroidogenic enzymes have been established in cell types throughout the brain, however the regulation of their expression is poorly understood (11, 31). In the adrenal glands of the HPA axis, IL-6 has been shown to increase the expression of both 11β-hydroxylase and 21-hydroxylase, leading to the increased production of CORT (10). IL-6 is one of several cytokines known to be elevated in the brain with age and contributes to the age-associated increase in inflammation (32, 33). IL-6 can act upon cells via classical or *trans*-signaling (34). Classical signaling involves IL-6 binding to its membrane bound receptor, which then dimerizes with the membrane bound gp130 receptor to elicit downstream signaling. The *trans*-signaling occurs when IL-6 binds to the soluble IL-6 receptor, followed by dimerization with membrane bound gp130. Several studies have implicated IL-6 *trans*-signaling as the major contributor to chronic inflammation with age as well as in neurodegenerative disease models (2, 34, 35).

The inhibition of *trans*-signaling is dependent upon the levels of the sgp130, which can bind to the soluble IL-6 receptor and prevent its signaling. Previous studies have shown that when sgp130 was administered to mice following lipopolysaccharide treatment, there was a significant reduction in inflammation in the brain as well as reduced sickness behavior (14). We reasoned that the age-related increase in IL-6 contributes to altered neurosteroid levels and reduced production of AlloP because: *a*) both IL-6 mRNA and protein levels increased in the cerebral cortex with age; and *b*) intracerebroventricular administration of IL-6 to young mice significantly reduced the levels of AlloP specifically in the cerebral cortex of male mice. Importantly, we also demonstrated that inhibition of IL-6 signaling via sgp130 intracerebroventricular infusion significantly improved spatial memory in aged mice and increased the levels of AlloP in the brain, although the latter effect failed to reach statistical significance.

Further studies are needed to determine whether the increased AlloP synthesis following IL-6 inhibition is part of the mechanism responsible for cognitive improvement. Previous studies indicate that both synapse number and function are reduced with age and contribute to cognitive impairments (36). To determine whether IL-6 inhibition affected synaptic abundance, we quantified the levels of the presynaptic marker, synaptophysin, and the post-synaptic marker, post-synaptic density protein 95 (PSD-95) in the hippocampus via Western blot. Using this approach, we observed a trend for a decrease with age, but the results were not significant, and there were no differences in the sgp130-treated groups. In addition, we quantified the astrocyte marker, GFAP, and the microglial marker, IBA-1, in the hippocampus to investigate whether IL-6 influenced the age-related increases in reactive astrocytes or microglia (37). Although there were trending increases in these markers with age, the results were not significant, and IL-6 inhibition did not alter GFAP or IBA-1 levels.

IL-6 is known to contribute to a variety of inflammatory pathways and likely has several downstream effects that may affect cognitive function directly, in addition to inhibiting AlloP synthesis (35). For example, chronic elevation of IL-6 in the brain has been shown to induce apoptosis, increase blood-brain barrier permeability, and induce neuronal excitotoxicity (38–40). All of these processes are associated with neurodegenerative diseases. Therefore, inhibition of IL-6 *trans*-signaling likely has multiple beneficial effects in the brain.

Our studies show that IL-6 administration resulted in a reduction in 5a-reductase expression, protein levels, and AlloP production specifically in astrocytes, rather than neurons. In contrast, IL-6 reduced 21-hydroxylase protein levels and reduced the production of CORT in neurons. Thus, the effect of IL-6 on steroidogenic enzymes is likely cell type specific, and interactions between multiple cell types likely have a key role in the cognitive actions of IL-6 (5). Although the use of primary cultures is an important and useful tool in assessing cell type-specific functions, it cannot recapitulate all of the complex changes that are evident in aged animals. Nevertheless, attempts were made to isolate astrocytes from the brains of aged mice, but, based on the viability of these cells, we were not convinced that the response of these cells would provide useful information. Future studies using acutely isolated astrocytes from aged mice would be useful to determine whether IL-6 specifically regulates AlloP synthesis in astrocytes, as we observed in our primary culture studies of embryonic mouse astrocytes.

Although the majority of cell types in the brain contain all the enzymes necessary to metabolize PRG into various neurosteroids, the relative levels of these enzymes likely differ (41). For instance, glial cells are thought to secrete more AlloP (42) than other cell types in the brain, thus the impact of IL-6 on AlloP production that we observe in vitro could also be occurring locally in the brain. Previous studies have shown that AlloP can increase hippocampal neurogenesis; therefore, its reduction with age is likely a contributing factor to the age-related decline in hippocampal neurogenesis.

Multiple factors are known to contribute to cognitive decline with age. These can include elevated inflammation, DNA damage, oxidative stress, and cellular senescence (3, 4, 43). It is possible that the reduction in AlloP with age is dependent on the elevation of IL-6 and is a major factor contributing to cognitive decline. Future studies are needed to determine the mechanism by which IL-6 downregulates 5α-reductase expression in the brain, as well as its effects on specific cell types throughout the brain.

### Data availability

All data reported in this study are located within the article.
